# The Use of Potassium Iodide in Pediatric Dentistry Does Not Change the Retention of Glass Ionomer Cement on a Dentin Treated with Silver Fluoride: In Vitro Results

**DOI:** 10.3390/dj12060177

**Published:** 2024-06-07

**Authors:** Louise Gotas, Thibault Canceill, Sabine Joniot

**Affiliations:** 1 Dental and Oral Medicine Department, Toulouse Universitary Hospital, 2 Rue de Viguerie, 31000 Toulouse, France; 2 Dental Department, Health Faculty of Toulouse, Toulouse III Paul Sabatier University, 3 Chemin des Maraichers, 31400 Toulouse, France; 3 Institut des Maladies Métaboliques et Cardiovasculaires (i2MC), Inserm/UPS UMR 1297, 1 Avenue Jean Poulhès, 31432 Toulouse, France

**Keywords:** pediatric dentistry, silver fluoride, glass ionomer cement, tooth restoration, dental materials

## Abstract

In pediatric and operative dentistry, caries treatment benefits from a therapeutic option based on the use of silver fluoride (AgF) associated with potassium iodide (KI) to avoid dark colorations on dental tissues. The objective of this in vitro study is to evaluate the retention of glass ionomer cement (GIC) on a dentin when treated with AgF and KI. Twenty-two healthy human permanent molars and eight human primary teeth, all free of any decay, were cut to obtain occlusal flat dentinal surfaces and were then treated with AgF for a duration of 60 s. For half of the teeth, a drop of KI was applied for a duration of 30 s. All samples were covered with a plot of GIC and their resistance to shear bond strength was measured. The fracture resistance in both permanent and primary teeth does not show any statistically significant differences whether KI was applied or not. For permanent teeth, the resistance is slightly higher in the group treated with KI than in the group treated with AgF alone. To our knowledge, these data are the first to describe the possible application of KI both on primary and permanent teeth. In any case, further studies are needed to investigate the bond strength between dentin and GIC on a wider range of samples.

## 1. Introduction

In pediatric and operative dentistry, caries treatment benefits from a therapy based on the use of silver diamine fluoride (SDF) to stop the processes of cavitation and bacterial proliferation [1]. This product is easy to handle and presents a long clinical history [2], recently modified with the availability of an aqueous solution of silver fluoride (AgF) that is free of ammoniac. However, the major weakness of these products is the dark coloration they cause on dental tissues [1]. To overcome this drawback, the application of potassium iodide (KI) in addition to AgF is proposed, but with limited effects according to the literature [3]. The whole treatment modality is called SMART, which stands for “Silver-Modified Atraumatic Restorative Treatment” [4]. Even if it cannot be occulted that the cure of a decay implied to eliminate infected tissues, for example with rotary instruments, these instruments does not seem to be mandatory. More than 20 years ago, Celiberti et al. highlighted the fact that manual instrumentation could represent the best option to eliminate a decay on a primary tooth in a child [5]. Over time, this concept has not been called into question and has even evolved, despite considerations of not eliminating all of the affected tissues [6]. Indeed, leaving a slice of affected dentin at the bottom of the cavity seems to reduce the risk of pulp aggression, as this tissue is able to be re-mineralized [7]. When accompanied by an adapted restoration, the total elimination of the affected dentin seems unnecessary to stop the carious lesion [8,9,10].

Historically, silver nitrate (AgNO_3_) was used in oral medicine [11,12] and was then replaced by silver diamine fluoride (Ag(NH_3_)_2_F_2_) during the second part of the twentieth century [13], at a concentration of 38%, i.e., 44,800 ppm of fluoride [14]. The first generations contained ammonia to stabilize the silver particles, but recently, some manufacturers have proposed products without ammoniac to avoid the unpleasant odor for the patient. The solution, with a basic pH around 10 [15], seems to present a real interest in stopping the evolution of a decay [16,17] and even in the treatment of tooth hypersensitivity [1,18].

SDF interacts with the hydroxyapatite of dental tissues according to the following chemical reaction: Ca_10_(PO_4_)_6_(OH)_2_ + Ag(NH_3_)_2_F_2_ → CaF_2_ + Ag_3_PO_4_ + NH_4_OH. Calcium fluoride and silver phosphate are created and can generate fluoroapatites in the case of an acidic pH [1,19]. Fluorine ions also have antibacterial effect and can penetrate in dentin tubules to avoid their colonization by microorganisms [20]; silver ions precipitate on the surface to limit the risk of secondary caries and to reduce the risk of post-operative sensitivities [21]. The depth of penetration for silver ions in tubuli remains, however, highly variable and discussed [22,23].

**Figure 1:**
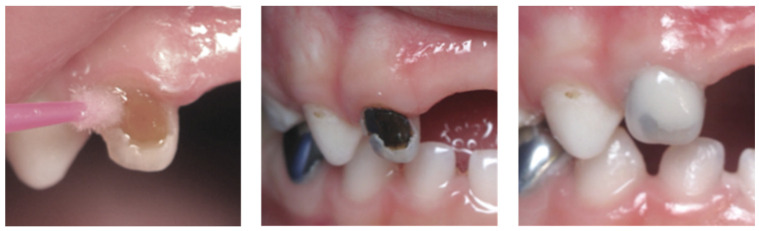
Treatment of a decay on tooth #52 in a 6-year-old boy with the application of silver diamine fluoride and glass ionomer cement. The picture in the center shows the aesthetic problem linked with the use of SDF.

Clinically, the minimum prerequisite before applying SDF or AgF is to eliminate unsupported debris in the cavity [17]; after application, the product and infected dentin can be immediately covered with a restorative biomaterial such as glass ionomer cement (GIC) (Figure 1), with a survival time estimated to be over 11 months [6]. However, despite its antibacterial effects, silver fluoride has deleterious aesthetic repercussions with a dark coloration of dental tissues [24]. In the evolution of protocols, the application of potassium iodide after that of AgF is now proposed to reduce the amount of dark coloring [4,25]. Indeed, potassium iodide (KI) creates a precipitate of tripotassium phosphate (K_3_PO_4_) and silver iodide (AgI), both in the form of white powders, according to the following reaction: Ag_3_PO_4_ + 3 KI → 3 AgI + K_3_PO_4_. There is, however, a lack of data concerning the possible consequences on the survival rate of restorative materials after the application of KI. 

The objective of this brief report is, thus, to evaluate the possible modification of glass ionomer cement retention on a dentin when treated with silver fluoride in combination with potassium iodide.

## 2. Materials and Methods

### 2.1. Study Design

An in vitro pilot study was performed in 2023 in the Health Department of Toulouse University (Département Odontologie, Faculté de Santé, Université Toulouse III—Paul Sabatier, Toulouse, France). It was conducted on two parallel groups, either with or without the application of potassium iodide as the only difference.

### 2.2. Tooth Preparation

Twenty-two human permanent molars extracted for orthodontic reason on patients aged under 20, as well as eight human primary molars, all free of any decay, restoration or fracture have been collected in accordance with the regulatory framework in force at our university hospital and displayed in patients’ waiting rooms (all healthcare waste may be collected for research purposes, in compliance with traceable storage conditions, unless the patient objects at the time of treatment). Once they were extracted, the teeth were placed in a 1% chloramine solution before being prepared for the study. The teeth were then plotted on poly-methylmethacrylate resin blocks (Ivolen, Ivoclar-Vivadent, Liechtenstein) in order to be easily cut with a low-speed diamond saw under irrigation (ISOMET^®^ Low Speed Saw, Buehler, ITW, IL, USA) to obtain occlusal flat dentinal surfaces. These surfaces were rinsed and dried; then, a drop of AgF was applied for a duration of 60 s (Riva Star Aqua Step 1, SDI, Australia). For half of the teeth (11 permanent and 4 primary teeth), a drop of potassium iodide (Riva Star Step2, SDI, Australia) was applied for a duration of 30 s. After this step, all the teeth were rinsed and slightly dried. A 20% polyacrylic acid solution was applied for a duration of 10 s and the surfaces were rinsed and slightly dried once again. The teeth were thus ready to receive their plot of glass ionomer cement (Riva Self Cure, SDI, Australia) (dimensions 2 mm height, 1.5 mm diameter).

### 2.3. Shear Bond Strength

All the samples were stored in a humid atmosphere at 37 °C for a duration of 48 h after the GIC were built up. Then, their resistance to shear bond strength was tested (UltraTester, Ultradent, UT, USA; conform to ISO 29022). A charge was applied from the device to the GIC plot, as close as possible to the tooth–material interface. The shear bond test was performed at a crosshead speed of 1 mm/min until the fracture of the glass ionomer cement plot. Once the GIC plot separated from the dentin, the tooth surface was observed under a binocular magnifier (zoom ×20) to determine the fracture mode. The rupture was classified as cohesive if it was localized only in the material or the tooth, or as adhesive if it was between the material and the tooth or as a mix of the two.

### 2.4. Statistical Analyses

Results were analyzed on Stata v13.0 software (Stata Corp, College Station, TX, USA) to compare the two groups (with or without the application of KI) with Mann–Whitney non-parametric tests for quantitative variables and with Fisher tests for fracture mode. The significant level was fixed at 5%.

## 3. Results

### 3.1. Shear Bond Strength

**Figure 2:**
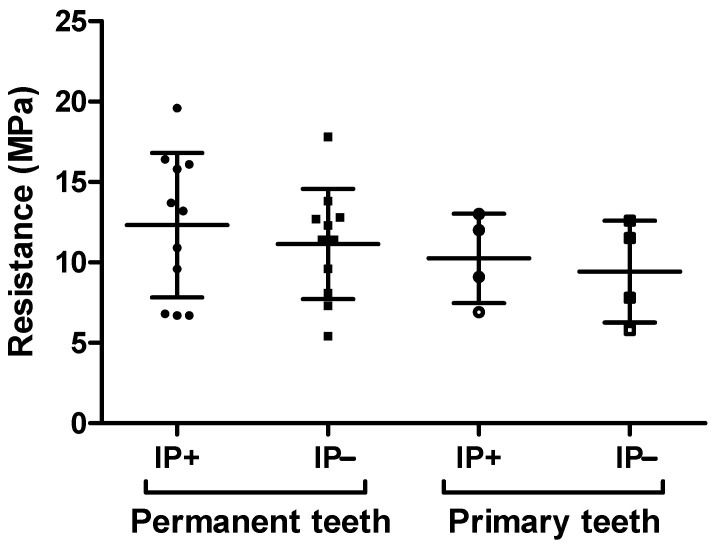
Scatter plot of all the samples submitted to shear bond strength. In each column, the main horizontal line indicates the mean, and the bar errors represent the standard deviation.

The fracture resistance in both permanent and primary teeth does not show any statistically significant difference whether or not potassium iodide was applied on the dentin. For permanent teeth, the resistance under shear stress is slightly superior in the group treated with potassium iodide (12.32 ± 4.49 MPa) than in the group treated with AgF alone (11.15 ± 3.43; *p* = 0.55). The trend is the same for primary teeth (10.25 ± 2.78 MPa with KI vs. 9.43 ± 3.17 MPa without KI; *p* = 0.56). The complete results with minimum and maximum values in each group are presented in Figure 2.

### 3.2. Fracture Mode

For all the primary teeth, fracture occurred between the dentin and the material, whereas for permanent teeth, a mixture of two fractures were noted in the group not treated with potassium iodide (Table 1).

## 4. Discussion

Our results highlight the absence of the impact of potassium iodide application on GIC resistance when used for tooth restoration. To our knowledge, even if these data have been obtained in vitro on a limited number of teeth, they are the first to study both primary and permanent teeth for the application of potassium iodide. In 2020, François et al. published results that match our own, with no differences concerning the resistance to shear stress as to whether potassium iodide was applied on dentin before high-viscosity GIC restorations or not [22]. However, Khor et al. have recently shown that resistance to the microtensile bond strength of glass ionomer cement was lower on a dentin treated with SDF in comparison to SDF-free tissues [26]. One possible explanation given by the authors for this difference is SDF’s basic pH, which may affect the efficiency of polyacrylic acid and the quality of the acid–base reaction existing inside the glass ionomer cement [26]. The authors also propose another hypothesis that incriminates dense precipitates such as calcium fluoride, which prevent ionic links between calcium bound to hydroxyapatite and GIC [26]. 

Concerning silver ions, even at low concentrations, they inhibit bacterial metabolism and reproduction by accumulating in the cell envelope, inducing the separation of the plasma membrane and the cell wall for both Gram+ and Gram- bacteria [27]. Mechanically, they seem to negatively affect the properties of a tooth–material interface because, as they compete with calcium ions in hydroxyapatite crystals, they limit the crosslinking between GIC and dentin [26]. The advantage of SDF and AgF is to combine the properties of silver ions and those of fluoride ions. This gives the products an effect against cariogenic bacteria such as *Streptococcus mutans*, whose colonization on the dentin surface is prevented in the presence of residual silver ions and fluorides [28]. The first authors to describe the persistent effect of these ions on bacterial lysis and the prevention of colonization on dental surfaces refer to this as a “zombie effect” [29]. Moreover, fluorine ions may reduce the pH inside the bacteria, which has an interest in inhibiting proteolytic enzymes that can alter the extracellular matrix and collagen [30]. 

In children, silver fluoride can be a future solution for the treatment of first molars affected by MIH (Molar Incisor Hypomineralization), a qualitative defect of the enamel structure affecting incisors and molars [31], leading to carious lesions that can rapidly lead to the extraction of the teeth. According to Schwendicke et al., approximately a quarter of the patients presenting with MIH need tooth restorative procedures. The simple application of AgF or SDF can thus constitute effective prevention against enamel caries, while also providing effective desensitization [32]. 

The risk of discoloration of the tooth can now be managed with potassium iodide, as proven in 2022 by Luong et al. [33], especially when the need for aesthetic is important on incisors and canines [4]. In the literature, the use of sodium hypochlorite has already been unsuccessfully tested in association with SDF, but silver ions are not sensitive to the effects of hypochlorite [25]. Studies that were carried out on the parental acceptance of treatments show that they accept the ease of application very well [34] and that the majority of parents are in agreement with a therapeutic solution that can delay a longer, more expansive and potentially more painful treatment, even if it is less aesthetic for their child [35]. 

Once the potassium iodide is applied, some modifications may be noted concerning the resistance of a future restorative material on dental tissues. For example, the application of SDF alone does not compromise the bonding values of composite resin restorations, whereas the association of SDF/KI weakens them [36]. This may be due to the formation of precipitates; thus, according to the American Food and Drug Administration, the issue in optimizing tooth restoration seems to lie in rinsing the tissues after the use of KI in order to eliminate the precipitates. With regard to the bonding of glass ionomer cement, studies show divergent results. In 2021, Sa’ada et al. concluded to a significantly greater shear strength for SDF-treated dentin than for dentin treated with an aqueous solution [37]; but, one year earlier, Uchil et al. found no difference between four treatments of decayed dentin on primary teeth (polyacrylic acid alone; 38% SDF alone; phosphoric acid etching and 38% SDF; and 38% SDF and 10% potassium iodide) [38]. In any case, further studies are needed to investigate the bond strength between dentin and GIC in the SMART treatment of primary teeth, on a wider range of samples.

The number of teeth used in this pilot study is, indeed, one of its limitations, as well as the absence of a control group made of GIC alone on the dentin surface. The decision to consider AgF treatment as a basic standard stems from the fact that our study focused on the SMART technique, i.e., the application of silver diamine fluoride on dentin. The interesting results, easily applicable to daily dental practice, can encourage the design of future studies, especially in clinically observing both the success and survival rates of glass ionomer cement restorations that are implemented on decayed teeth in children and adults.

## 5. Conclusions

Increasingly being re-used, silver diamine fluoride is a therapeutic solution of choice in pediatric dentistry, offering a safe, effective and inexpensive alternative to more invasive conventional care in the treatment of carious disease. Our pilot study shows the possibility to apply SDF in combination with potassium iodide, before restoring the tooth with a glass ionomer cement. 

## Figures and Tables

**Table 1:** Fracture mode under shear bond strength obtained on primary and permanent teeth.

	Fracture Mode	Cohesive	Adhesive	Mixture	*p*-Value
Primary teeth	IP+ group (n = 4)	0	4 (100%)	0	>0.99
	IP− group (n = 4)	0	4 (100%)	0	
Permanent teeth	IP+ group (n = 11)	0	11 (100%)	0	0.47
	IP− group (n = 11)	0	9 (81.8%)	2 (18.2%)	

## Data Availability

Data are available upon reasonable request to the authors.
